# ERBB2D16 Expression in HER2 Positive Gastric Cancer Is Associated With Resistance to Trastuzumab

**DOI:** 10.3389/fonc.2022.855308

**Published:** 2022-04-07

**Authors:** Shuo Wang, Yuze Zhao, Yuguang Song, Guoliang Qiao, Yan Di, Jing Zhao, Pingping Sun, Huixia Zheng, He Huang, Hongyan Huang

**Affiliations:** ^1^ Department of Oncology, Beijing Shijitan Hospital, Capital Medical University, Beijing, China; ^2^ Department of Surgical Oncology, Massachusetts General Hospital, Boston, MA, United States; ^3^ Department of Pathology, Beijing Shijitan Hospital, Capital Medical University, Beijing, China; ^4^ Department of Pathology, First Hospital of Shanxi Medical University, Taiyuan, China; ^5^ Department of General Surgery, First Hospital of Shanxi Medical University, Taiyuan, China

**Keywords:** gastric cancer, ERBB2d16, epithelial-mesenchymal transition, immunosuppression, trastuzumab resistance

## Abstract

The human epidermal growth factor receptor-2 (ERBB2; formerly HER2)isoform ERBB2ΔEx16 (ERBB2d16) was oncogenic by mediating epithelial-mesenchymal transition (EMT), immune evasion, and resistance cell death to the anti-HER2 (trastuzumab) therapy. However, its physiological implications in gastric cancer were unclear. In this study, we examined a total of 110 patients with either locally advanced or metastatic HER2^+^ gastric cancer for the expression of ERBB2d16 and EMT markers, and the infiltration of CD3^+^ T cells in tumor tissues, and evaluated their relevance with the responses to the standard chemotherapy plus trastuzumab according to the RECIST criteria. We found that the ERBB2d16 isoform was present at a relatively high level in about half of the tumor samples examined (53/110) and an elevated ERBB2d16/ERBB2 ratio was positively associated with the expression of high E-cadherin and low vimentin indicating EMT, and with poor CD3+ T cell infiltration and strong intratumoral expression of programmed death 1 (PD-1) and programmed death ligand 1 (PD-L1) as well as reduced diversity of T cell receptor clones. Moreover, the progression-free survival and overall survival of patients treated with trastuzumab were substantially shorter in those with a high ERBB2d16/ERBB2 ratio. In agreement, analysis by Cox proportional hazards models confirmed that high ERBB2d16 expression was a risk factor associated with an adverse prognosis. Thus, our data fit well with an oncogenic role of ERBB2d16 in gastric cancer by promoting EMT and immunosuppression. We also found that ERBB2d16 expression resists gastric cell death in patients treated with trustuzumab, and the ERBB2d16/ERBB2 ratio may serve as a novel prognostic maker for patients with gastric cancer that receive trastuzumab therapy.

## Introduction

Gastric and gastroesophageal junction (G/GEJ) adenocarcinomas are the 4th leading cause of cancer-related death worldwide (1). Overexpression of ERBB2, a marker of highly aggressive tumors, is observed in 20-23% of G/GEJ adenocarcinomas. Treatment with the anti-HER2 antibody trastuzumab combined with platinum-containing chemotherapy combinations improves progression-free and overall survival in advanced HER2-overexpressing G/GEJ adenocarcinoma (2); however, resistance to the therapy eventually develops, although the mechanisms are poorly understood (3–6).

**Figure 1:**
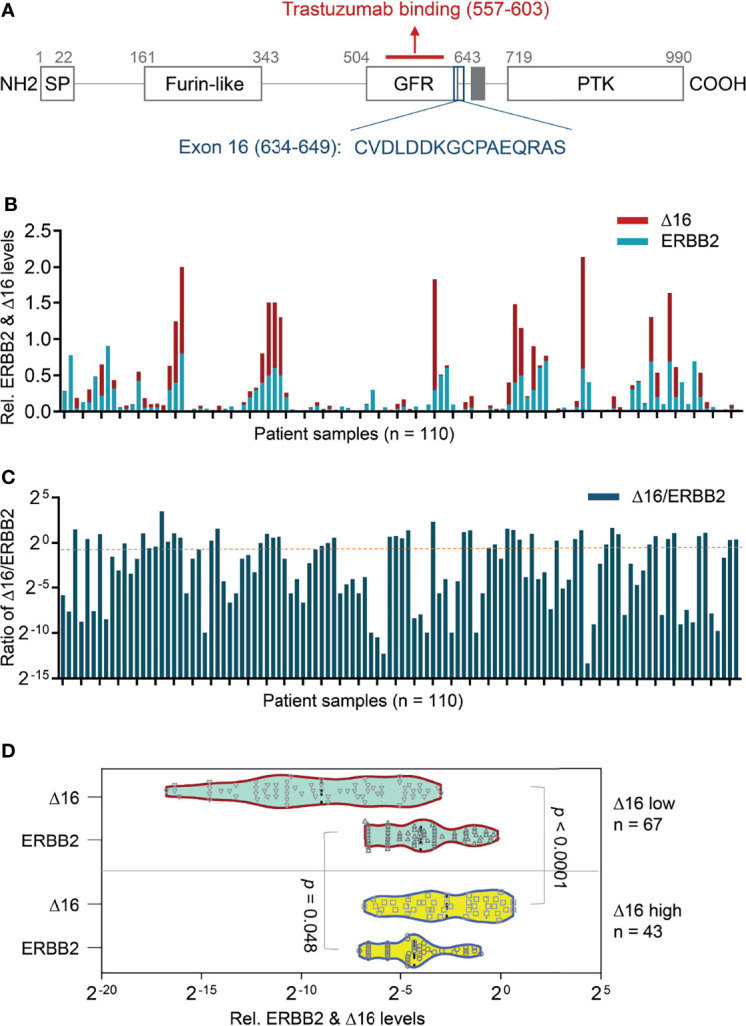
Transcriptional expression of ERBB2d16 and ERBB2 in gastric cancer. **(A)** The schematic structure of the ERBB2d16 protein; **(B)** The transcriptional expression of ERBB2 and ERBB2d16 by quantitative RT-PCR in gastric cancer tissue with beta-actin as an internal control (n = 110); **(C)** The ratios of the ERBB2d16/ERBB2 in HER2-positive gastric cancer tissues (n = 110). **(D)** An ERBB2d16/ERBB2 ratio-dependent expression pattern of ERBB2d16 relative to ERBB2 expression. Δ16 low: ERBB2d16/ERBB2 ratio < 0.88; Δ16 high: ERBB2d16/ERBB2 ratio ≥ 0.88. *ERBB2: human epidermal growth factor receptor-2; ERBB2d16: ERBB2ΔEx16; RT-PCR: reverse transcriptive polymerase chain reaction*.

In HER2-overexpressing breast cancer, one emerging explanation for resistance to trastuzumab is the presence of the ERBB2d16 isoform, which contains a 16-amino-acid in-frame deletion in the juxta transmembrane domain (**Figure 1A**). Initial evidence suggests that ERBB2d16 promotes tumor invasion and metastasis and resistance to trastuzumab (7). Furthermore, the notch pathway is highly activated in the presence of ERBB2d16 but not in the presence of full-length ERBB2; thus, ERBB2d16 expression is associated with stemness and EMT (8), both of which are related to therapeutic resistance.

As some reports have confirmed that tumors exhibiting EMT markers also demonstrate immunosuppressive features [including low levels of class I major histocompatibility complex (MHC-I), high levels of PD-L1 and PD-1 (9) and stromal infiltration of regulatory T cells, M2 (protumor) macrophages, and exhausted CD8+ T cells (9, 10)]. The diversity of the T cell receptor (TCR) repertoire correlates with the presence of tumor neoantigen-specific T cells (11). When a tumor occurs, many new antigens are produced by mutations in the gene, the production of some new antigen stimulates T cells with TCR that recognize this neoantigen, and then amplified itself and killed tumor cells. By sequencing the V (D) J region in the TCR ecosystem, we can indirectly understand the immune micro-environment around the tumor (12).

Considering the role of ERBB2d16 in the therapeutic resistance of HER2-overexpressing G/GEJ adenocarcinomas has not been well described, in the present study, we analyzed the expression of ERBB2d16 and markers of EMT (E-cadherin, vimentin) in tumors from 110 patients with HER2 positive gastric cancer who had been treated with standard chemotherapy plus trastuzumab and explored the correlation of ERBB2d16 expression with EMT, intratumoral infiltration of immune cells along with TCR repertoire diversity, and prognosis.

## Materials and Methods

### Collection of Samples and Clinical Data

All participants provided written informed consent for the use of their specimens and clinical data in this research. These specimens were formalin-fixed, paraffin-embedded (FFPE) gastric cancer specimens collected as part of standard-of-care gastrectomy procedures from patients subsequently treated with at least one line of conventional chemotherapy plus trastuzumab between February 2017 and January 2020 in Beijing Shijitan Hospital and First Hospital of Shanxi Medical University. The study protocol was approved by the Institutional Review Board of Beijing Shijitan Hospital Ethics Committee, and the ethics number is sjtk11-1x-2021 (34) and (2019) (SK014). All patients were treated in accordance with the Declaration of Helsinki. All patients were confirmed to have HER2-overexpressing gastric cancer using the IHC method, and HER2++ status was confirmed by the fluorescence *in situ* hybridization (FISH) method. Patient characteristics (sex, age, stage at the time of diagnosis according to the eighth edition of the American Joint Committee on Cancer [AJCC] tumor-node-metastasis [TNM] staging system (13), alcohol use and smoking status), tumor characteristics (histology, primary localization and the number of metastatic sites, site in stomach, cardia involvement status and H. pylori status), number of previous chemotherapy lines, date of initiation of trastuzumab and date of death were extracted from patient medical records (**Table 1**).

**Table 1:** Patient and tumor characteristics (N = 110).

Characteristics	No. of cases (n, %)	ERBB2d16 High	ERBB2d16 Low	*P*-value
		n = 43 (39%)	n = 67 (61%)	
**Age, years**				
Median (range)	55 (30-81)	53 (30~61)	56 (41~81)	0.82
**Sex**				
Male	58 (53%)	26 (60%)	32 (48%)	0.75
Female	52 (47%)	17 (40%)	35 (52%)	
**Differentiation grade**				
Poor-undifferentiated	83 (75%)	34 (79%)	49 (73%)	0.093
Well-moderate	27 (25%)	9 (21%)	18 (27%)	
**TNM stage**				
I-III	98 (89%)	35 (81%)	63 (96%)	0.051
IV	12 (11%)	8 (19%)	4 (4%)	
**Site of tumor**				
Body of stomach	100 (91%)	39(91%)	61 (91%)	0.87
Cardia	10 (9%)	4 (9%)	6 (9%)	
**Number of metastatic lesions**				
0	98 (89%)	35 (81%)	63 (94%)	0.51
1	5.(5%)	2 (5%)	3 (4%)	0.29
≥2	7 (6%)	6 (14%)	1 (2%)	0.36
**Chemotherapy**				
CapeOX	48 (47%)	19 (44%)	29 (43%)	0.66
SOX	39 (29%)	15 (35%)	24 (36%)	0.14
Others*	23 (24%)	9 (21%)	14 (21%)	0.24
**Line of treatment**				
1	33 (30%)	12 (28%)	21 (31%)	0.68
2	14 (13%)	5 (12%)	9 (13%)	0.91
≥3	63 (57%)	26 (60%)	37 (55%)	0.27
**Smoking history**				
Yes	61 (55%)	24 (56%)	37 (55%)	0.51
No	49 (45%)	19 (44%)	30 (45%)	
**Drinking history**				
Yes	52 (47%)	19 (44%)	33 (49%)	0.52
No	58 (53%)	24 (56%)	34 (51%)	
**Helicobacter Pylori infection**				
Yes	90 (82%)	35 (81%)	55 (82%)	0.44
No	20 (8%)	8 (9%)	12 (18%)	

CapeOX ,capecitabine+oxaliplatin; SOX, S1+ oxaliplatin; *Capecitabine (n = 5); FOLFOX (n = 8); Cisplatin+5-Fu (n = 6); Cisplatin+ capecitabine (n = 4).

### Extraction of Total RNA

FFPE tissue blocks were sectioned on a standard microtome (Leica Microsystems RM 2145) to generate successive 10-μm sections. A pathologist evaluated each slide and confirmed and marked regions of invasive carcinoma. For each sample, marked regions from several slides (number depending on the size of the invasive area, according to the manufacturer’s instructions) were microdissected using a new sterile blade, and the dissected tissues were placed immediately into an RNase-free microcentrifuge tube. After deparaffinization with xylene, the tissues were digested with protease and treated with DNase. After washing, the RNA, including the small microRNA fraction, was eluted with 60 μl distilled water. The concentration and quality of the RNA recovered were measured using a Nanodrop 2000 spectrophotometer (Nanodrop Technologies, Wilmington, DE, USA). The median 260/280 ratio was 2.05 (interquartile range [IQR]: 2.01–2.1), and the median concentration was 246.27 ng/μl (IQR: 120.5–455.5).

### Primers

For polymerase chain reaction (PCR) amplification of ERBB2d16, ERBB2, E-cadherin, vimentin, PD-1, PD-L1 and the reference gene β-actin, primers were designed using Primer Premier 5.0 software (Applied Biosystem, USA). See **Table 2** for details.

**Table 2:** Prime sequence.

Prime	Forward	Reverse
ERBB2	5’-CTGCACCCACTCCTGTGTGGACCTG	5’-CTGCCGTCGCTTGATGAGGATC
ERBB2d16	5’-CTGCACCCACTCCCCTCTGAC	5’-CTGCCGTCGCTTGATGAGGATC
E-cadherin	5’-CGAGAGCTACACGTTCACGG	5’-GGGTGTCGAGGGAAAAATAGG
Vimentin	5’-GACGCCATCAACACCGAGTT	5’-CTTTGTCGTTGGTTAGCTGGT
PD-1	5′-CGTGGCCTATCCACTCCTCA	5’-ATCCCTTGTCCCAGCCACTC
PD-L1	5′- AAATGGAACCTGGCGAAAGC	5′- GATGAGCCCCTCAGGCATTT
β actin	5′- TTAGTTGCGTTACACCCTTTC	5′- ACCTTCACCGTTCCAGTTT

ERBB2, human epidermal growth factor receptor-2; ERBB2d16, ERBB2ΔEx16; PD-1, programmed death 1; PD-L1, programmed death ligand 1.

### Reverse Transcription-PCR Analysis

Total RNA was converted to cDNA using Maxima H Minus cDNA Synthesis Master Mix with ds DNase (Thermo Scientific MAN0016393, USA). Quantitative PCR was performed in triplicate using 1/10 of the RT product with the Power SYBR Green RNA-to-CT 1-Step Kit (Applied Biosystems A25742). All samples were analyzed in triplicate with error bars representing SD. We calculated the differences in expression amount according to CT values of each sample using 2-ΔCT to define the differences in gene expression among samples. β-Actin was used as the internal reference.

### Immunofluorescence Staining and Antibody

We selected 20 patients with the highest ERBB2d16/ERBB2 ratio (ERBB2d16 high) and 20 patients with the lowest ERBB2d16/ERBB2 ratio (ERBB2d16 low) for immunofluorescence staining. For Histo-Cytometry (Multiplex Quantitative Tissue Imaging Analysis), 10-μm sections from the gastrectomy FFPE tissue blocks were loaded onto slides, deparaffinized in xylene, and rehydrated in a series of graded alcohols. Antigen retrieval was performed in citrate buffer (pH 6) in a microwave (Sharp, R-331ZX) for 20 min at 95°C followed by a 20-min cool down at room temperature. After quenching endogenous peroxidase in 3% H2O2, the slides were incubated with a blocking reagent (ZSGB-BIO, ZLI-9022) for 30 min at room temperature. Antigens were then successively detected using the Opal protocol (14, 15). Briefly, the sections were incubated with the primary antibody for 120 min in a humidified chamber at 37°C, followed by detection using an HRP-conjugated secondary antibody (GBI Labs, Polink-1 HRP polymer detection kit) and TSA-fluors (PerkinElmer, Opal 7-color IHC Kit, NEL797001KT, 1:100, 20–60 sec), after which the primary and secondary antibodies were thoroughly eliminated by heating the slides in citrate buffer (pH 6.0) for 10 min at 95°C in a microwave. Serially, each antigen was labeled by distinct fluorophores. The nuclei were subsequently visualized with DAPI (1:2000), and a coverslip was applied using Prolong Gold Antifade Mountant (Thermo Fisher, P36934). The multiplex antibodies applied in this study were as follows: anti-CD3 (Abnova MAB9626, 1:100, Opal 690), PD-1 (CST, #43248, 1:200, Opal 620), PD-L1 (CST, #13684, 1:200, Opal 650), E-cadherin (CST, #3195, 1:500, Opal 650), and anti-vimentin (Abcam, ab137321,1:400, Opal 570).

### Digital Image Acquisition and Analysis

We used a TissueFAXS slide scanning system (Tissue Gnostics) based on the Zeiss Axio Imager Z2 vertical epifluorescence microscope to scan the fluorescently labeled slides. At the same time, we used a Zeiss 20 Plan Apochromat air objective (0.8 NA) to capture the image. Tissue Quest software (Tissue Gnostics) was used to analyze the fluorescence values. The DAPI-stained nuclei were segmented, and thresholds set for different intensities were used to identify all DAPI-stained nuclei. We used the Polaris imaging system to scan the whole section of the samples with low magnification (4X), then selected the representative fields of views (10-15) from the sample panorama, scanned with 20X, and then did sample analysis. The software provided with Polaris system was used to distinguish the tumor and the stromal area through the method of tissue self-learning, and then the tissue and stromal cells were identified by the DAPI channel. Based on the intensity of the fluorescent signal of each cell, the software identified positive cells and counted the number of positive cells for a specific phenotype. For each sample, the sum of the number of positive cells in all fields of a view was divided by the sum of the areas of the tissue regions corresponding to all fields of the view to obtain the cell density of the specific cell phenotype for each sample (16).

### T Cell Receptor (TCR) Sequencing

For TCR sequencing, twenty patients with the highest ERBB2d16/ERBB2 ratio (ERBB2d16 high) and 20 patients with the lowest ERBB2d16/ERBB2 ratio (ERBB2d16 low) were selected and 10 ml peripheral blood sample was collected before treatment for metastatic or relapsed disease. Then the DNA was extracted by using a Qiagen DNA blood kit, or DNA blood mini kit (Qiagen).

Complementarity determining region-3 (CDR3) in the TCR β chain (TRB) was inclusively and semiquantitatively amplified by multiplex PCR; the multiplex amplification included both first and second rounds of PCR (PCR1 and PCR2). The primer sequences have been filed as a part of a Chinese patent (CN105087789A). During the first round of PCR (PCR1), 10 cycles were used to amplify CDR3 sequences using specific primers for each V and J gene. In the second round of PCR, PCR was performed using universal primers. For PCR1, template DNA (600 ng) was amplified after adding 2× QIAGEN Multiplex PCR Master Mix (25 µl), 5× Q solution (5 µl), the forward primer set pool (1 μl), and the reverse primer set pool (1 μl) to make a reaction system by using a Multiplex PCR Kit (QIAGEN, Germany). Then, PCR was performed with 1 cycle of 95°C for 15 min, 10 cycles of denaturation at 94°C for 30 seconds, and 10 cycles of annealing at 60°C for 90 seconds and extension for 30 seconds at 72°C. After a final extension for 5 min at 72°C, the system was cooled to 4°C. The multiplex PCR products was purified with magnetic beads (Agencourt no. A63882, Beckman, Beverly, MA, USA). All PCR1 products were used as templates for the second step of amplification after adding pooled primers (2 µl), Phusion master mix prepared using a Phusion^®^ High-Fidelity PCR Kit (25 µl; New England Biolabs, America), and nuclease-free water to reach a total volume of 50 µl. The reactions were then transferred to a thermal cycler that carried out the following program: one cycle at 98°C for 1 min; 25 cycles of denaturation at 98°C for 20 seconds, annealing at 65°C for 30 seconds and extension at 72°C for 30 seconds; and a final extension at 72°C for 5 min. The samples were then held at 4°C. Size selection was performed by agarose gel electrophoresis (400 mA/100 V, 2 h), and the targeted fragments (between 200-350 bp) were retrieved and purified by a QIAquick Gel Purification Kit (QIAGEN, Germany). The paired-end sequencing of these samples was carried out with a read length of 151 bp using an Illumina HiSeq 3000 platform (17).

Raw sequencing data were processed and analyzed as follows. 1) Undesired sequences that did not contain the primers were filtered using Cutadapt (https://cutadapt.readthedocs.org/), 2) reads were merged to obtain contigs using Pear (https://cme.h-its.org/exelixis/web/software/pear/doc.html), 3) sequences were aligned with the reference TRB V/(D)/J gene sequences (http://www.imgt.org) using MiXCR to determine the TRB V/(D)/J gene segment in each contig, 4) the CDR3 region was identified based on the conserved sequence of the CDR3 region, and 5) CDR3 species were clustered to eliminate sequencing errors according to the base quality and sequence similarity (18).

### Interpretation of Immune Repertoire Analysis Results

In this study, Shannon index for diversity and clonality index along with evenness index were used to exhibit the characteristics of immune repertoire as previously reported (19–21). The diversity of the TCR repertoire was calculated based on the Shannon–Wiener index (Shannon index), which is a function of both the relative number of clonotypes presence and the relative abundance or distribution of each clonotype (19). The Shannon index is calculated as follows. In the Shannon index, “ni” is the clonal size of the clonotype (that is, the copies of a specific clonotype), “s” is the number of different clonotypes, and “n” is the total number of TCR/B cell receptor (BCR) sequences analyzed.


$$Shannonindex=-\sum_{i=1}^{s}\frac{ni}{N}ln\frac{ni}{n}$$

Clonality index is defined as 1- (Shannon index)/ln(# of productive unique sequences) (20). A maximally diverse population is associated with a clonal score of 0, and a perfect monoclonal population is associated with a clonality score of 1.

Mathematically, the evenness index is defined as a diversity index, similar to the clonicity index (21). Evenness measured the homogeneity of clones. The higher Evenness value was, the more evenly distributed the clones were. The lower Evenness value was, the more oligo-clones were amplified.

### Clinical Outcome Data

Clinical and laboratory examinations were carried out within 7 days before enrollment and each cycle of chemotherapy afterward. Tumor measurement was conducted on the basis of computed tomographic scans, within 15 days before enrollment and every 3 months in the admission. Discontinuation of therapy occurred in the event of progression of the disease, patient refusal, unacceptable toxicity, or death. Overall Survival (OS) was defined as the period from the date of first treatment until death. Patients who did not experience an event were censored on the date of the last contact. Progression-free survival (PFS) was measured from the day that chemotherapy plus trastuzumab was initiated until progression of disease or death was documented.

### Statistical Analysis

Continuous variables were expressed as mean ± SD (standard deviation) and compared using a two-tailed unpaired Student’s t-test; categorical variables were compared using χ2 or Fisher analysis. The correlations were measured by calculating the Pearson’s correlation coefficient (r). Life-table estimates of survival time were calculated for the evaluation of PFS and OS as the primary end-point, according to the Kaplan and Meier methodology. A Cox proportional hazards regression approach was chosen for the evaluation of PFS and OS as the primary end-point. All statistical evaluations were carried out using GraphPad Prism (GraphPad Software, version 8.0) and SPSS software (Statistical Package for the Social Science, version 15.0, SPSS Inc.). (*: P<0.05; **: P<0.001; ***: P<0.0001; ns: not significant).

## Results

### Expression of ERBB2d16 and ERBB2 in Gastric Cancer

The expression of ERBB2 and ERBB2d16 was examined using quantitative RT-PCR in the pretreatment gastrectomy specimens of 110 patients with HER2-overexpressing gastric cancer. As shown in **Figure 1B**, both ERBB2 and ERBB2d16 were detectable in each specimen, the ERBB2d16 isoform was present at a relatively high level in about half of the tumor samples examined (53/110). Here, a ratio value (Ct d16/Ct actin) of 0.02 was set as the cut-off for high and low expression. Whereas, the level of ERBB2d16 relative to that of ERBB2 varied substantially, as previously reported in breast cancer specimens. To standardize the expression level of ERBB2d16, the ratio of the expression of ERBB2d16 to the expression of ERBB2 (ERBB2d16/ERBB2) was calculated and used to present the relative expression level of ERBB2d16 with a defined cutoff as 0.88 (**Figure 1C**). Patients were then stratified into two groups according to the ratio (ERBB2d16/ERBB2) with a cutoff value of 0.88 derived from ROC curve, one group (ERBB2d16 low expression) included 67 patients with the ratios less than 0.88 and the other group (ERBB2d16 high expression) included 43 patients with the ratios no less than 0.88. The expression of ERBB2 and ERBB2d16 all exhibited significant differences between the two groups (**Figure 1D**).

Detailed clinical information for all patients was shown in **Table 1**. The result of univariate analysis demonstrated that ERBB2d16 expression was not associated with known clinicopathological features, including patient age, gender, smoking or drinking history, adjuvant chemotherapy, H. pylori status, tumor location, the differentiation and TNM stage of the tumor.

### Correlation of an Elevated ERBB2d16/ERBB2 Ratio With the EMT-Like Phenotype

**Figure 2:**
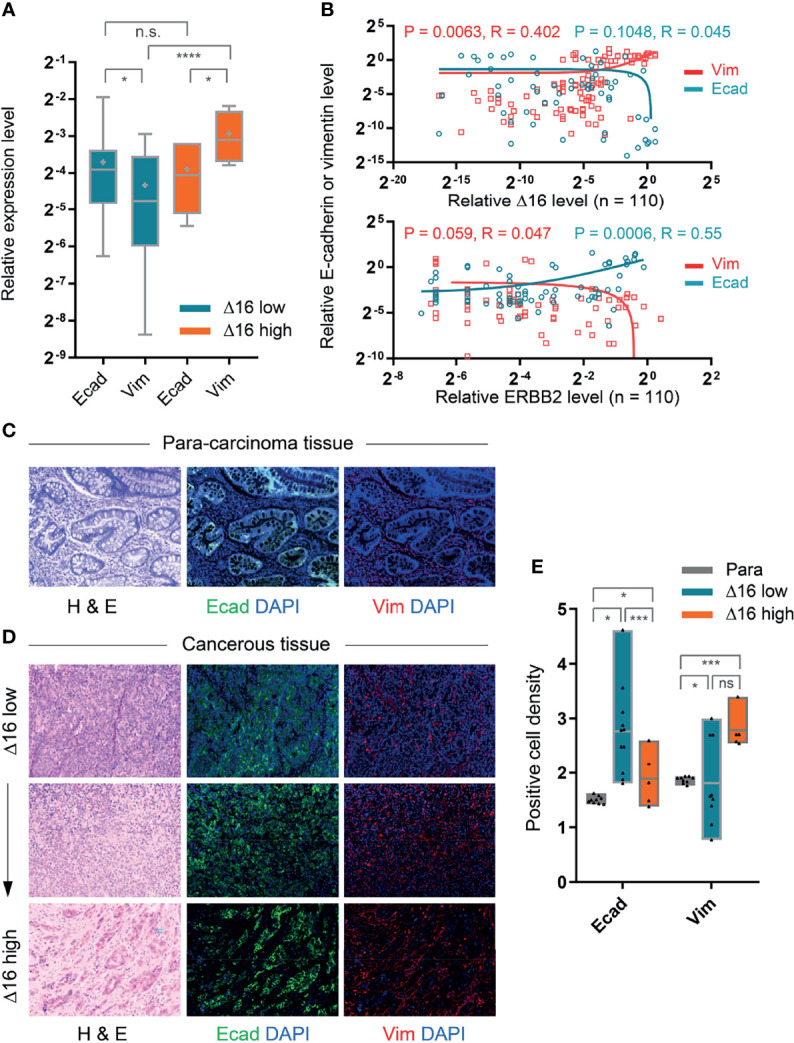
Correlation of high ERBB2d16 expression with EMT-like phenotype. **(A)** Relative mRNA expression levels of E-cadherin (Ecad) and vimentin (Vim) in ERBB2d16 (Δ16) -low (n = 67) and -high (n = 43) groups, respectively. **(B)** The correlations between ERBB2 isoforms and EMT markers (E-cadherin and vimentin) by the Pearson’s correlation analysis (n = 110); **(C, D)**. Hematoxylin and eosin (H&E) and immunofluorescent staining (E-cadherin and vimentin) of paired para-carcinoma tissue **(C)** and tumor tissues **(D)**. Scale bar, 100 μm; **(E)**. The expression of E-cadherin and vimentin, which was represented by the density of positive cells, in tumor tissue of low ERBB2d16 (n = 20), of high ERBB2d16 (n = 20), and paired para-carcinoma tissues (n = 40). (*p < 0.05, **p < 0.001, ***p < 0.0001, ****p < 0.00001), ns, not significant. Δ16 low: ERBB2d16/ERBB2 ratio < 0.88; Δ16 high: ERBB2d16/ERBB2 ratio ≥ 0.88. *ERBB2: human epidermal growth factor receptor-2; ERBB2d16: ERBB2ΔEx16*.

To explore the relationship between ERBB2d16 and EMT in tumor tissue samples, we performed quantitative RT-PCR to examine the difference in gene expression levels of E-cadherin and vimentin between the high and low ERBB2d16 groups. As shown in **Figure 2A**, the expression of vimentin in the high ERBB2d16 expression group was significantly higher than that in the low ERBB2d16 expression group (P < 0.0001). However, the expression of E-cadherin was not significantly different between the high and low ERBB2d16 groups (P = 0.093). The expression of E-cadherin was significantly higher than that of vimentin in the low ERBB2d16 expression group (P = 0.04), whereas the expression of vimentin was higher than that of E-cadherin in the high ERBB2d16 expression group (P = 0.014). This result suggested that the increase in ERBB2d16 may mainly affect vimentin expression.

Furthermore, Pearson linear regression was then performed to analyze the correlation between ERBB2 or ERBB2d16 expression and E-cadherin or vimentin expression. The result indicated that as the expression of ERBB2 increased, the expression of E-cadherin increased accordingly while expression of vimentin decreased, suggesting an inverse correlation (**Figure 2B**). In contrast, as ERBB2d16 expression increased, the expression of vimentin increased while the expression of E-cadherin decreased. Therefore, the results suggested that the elevated ERBB2d16 might be associated with EMT.

We also validated these results at the protein level using multitarget immunofluorescence staining to label E-cadherin and vimentin in the patient tumor tissues and the paired para-carcinomous tissues with fluorescently labeled antibodies (**Figures 2C, D**). The fluorescence intensity of each pixel point on a microscopy image reflects the expression level of the targeted protein such that the mean of the total number of fluorescence values in an image reflects the corresponding mean protein expression level. As shown in **Figure 2E**, the expression of E-cadherin or vimentin was represented by the density of positive cells. We found that expression of vimentin was significantly higher in tumor tissues with high ERBB2d16 expression than in the surrounding nonmalignant tissues, while the expression of E-cadherin was lower; in contrast, the expression of vimentin was similar in tissues with low ERBB2d16 expression and the surrounding, nonmalignant tissues, while E-cadherin was significantly higher than in the surrounding, nonmalignant tissues. Additionally, we noticed that the expression level of vimentin was different between the ERBB2d16 low and high groups although the difference was not statistically significant. As far as the difference of vimentin expression between ERBB2d16 high and low groups, it was also reflected partially by the density of vimentin-positive cancer cells. Meanwhile, the vimentin-positive stromal cells were present in both ERBB2d16 high and low groups, which may largely compromise the differences caused by EMT in cancer cells.

### Association of an Elevated ERBB2d16/ERBB2 Ratio With Immunosuppressive Microenvironment

**Figure 3:**
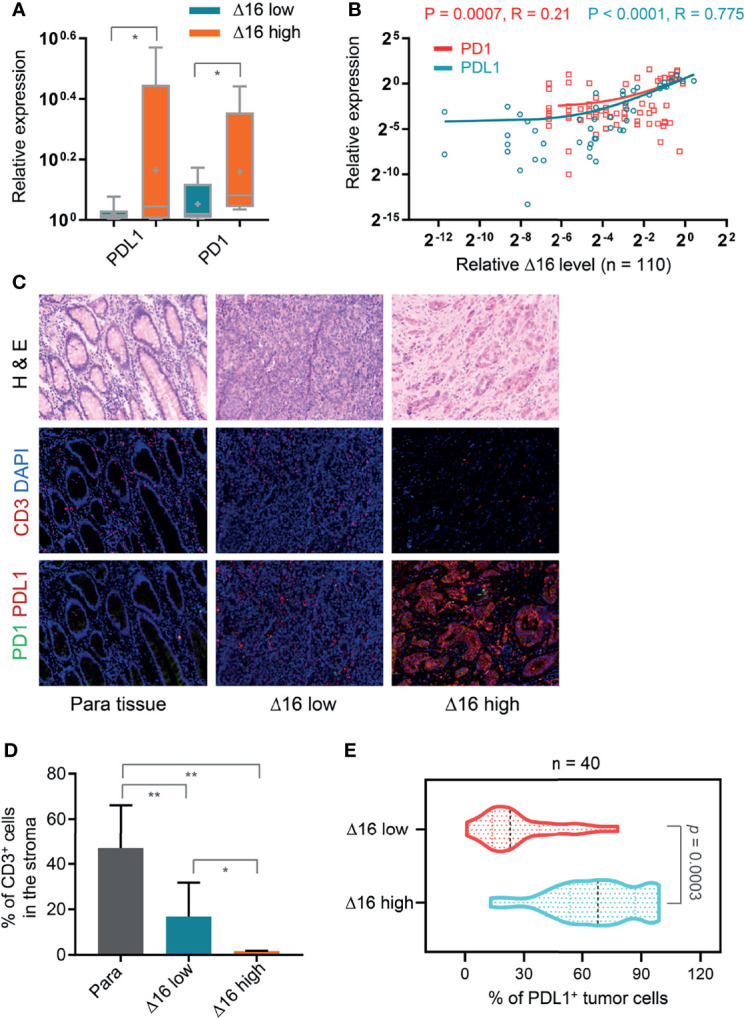
Association of an elevated ERBB2d16/ERBB2 ratio with immunosuppressive microenvironment. **(A)** Relative mRNA expression levels of PD-L1 and PD-1 in ERBB2d16 (Δ16) -low (n = 67) and -high (n = 43) groups, respectively. **(B)** ERBB2d16 expression is positively correlated with those of PD-1 and PD-L1 as determined by Pearson’s correlation analysis (n = 110). **(C)** Hematoxylin and eosin (H&E) and immunofluorescent staining (CD3, PD-1 and PD-L1) of tumor tissues and the paired para-carcinoma tissues. Scale bar, 100 μm; **(D)** The percentage of the CD3-positive cells in the stroma of ERBB2d16-high (n = 20), and -low tumor tissue (n = 20), and the paired para-carcinoma tissue (n = 40), respectively. **(E)** The percentage of PD-L1-positive tumor cells in ERBB2d16-high (n = 20), and -low tumor tissue (n = 20) tumor tissues, respectively. (*p < 0.05, **p < 0.001, ***p < 0.0001). Δ16 low: ERBB2d16/ERBB2 ratio < 0.88; Δ16 high: ERBB2d16/ERBB2 ratio ≥ 0.88. *ERBB2: human epidermal growth factor receptor-2; ERBB2d16: ERBB2ΔEx16; PD-1: programmed death 1; PD-L1: programmed death ligand 1*.

Because of previous observation that EMT is associated with an immunosuppressive environment, we evaluated the relationship of ERBB2d16 expression and the immune markers PD-1 and PD-L1 at the gene expression level and intratumoral infiltration with CD3+ T cells. As shown in **Figure 3A**, tumors with high ERBB2d16 levels had higher PD-1 and PD-L1 levels, and the expression levels of these molecules tended to be correlated with those of ERBB2d16 (**Figure 3B**). In addition, the highest number of T cells was observed in the nonmalignant surrounding tissue; however, the number of CD3+ T cells in samples with low ERBB2d16 expression was significantly higher than that in the samples with high ERBB2d16 expression (P=0.038, **Figure 3D**), and as the expression level of ERBB2d16 increased, the PD-L1 expression increased concomitantly (P=0.0003, **Figure 3E**). We confirmed the findings of the gene expression analyses with immunofluorescence staining for CD3 and PD-L1. PD-L1-expressing cells accumulated in patients with high ERBB2d16-expressing tumors (**Figure 3C**). Taken together, these data suggest that tumors with high ERBB2d16 expression might have immunosuppressive microenvironments.

### Reduced Diversity of TCR Clones in ERBB2d16 High Patients

**Figure 4:**
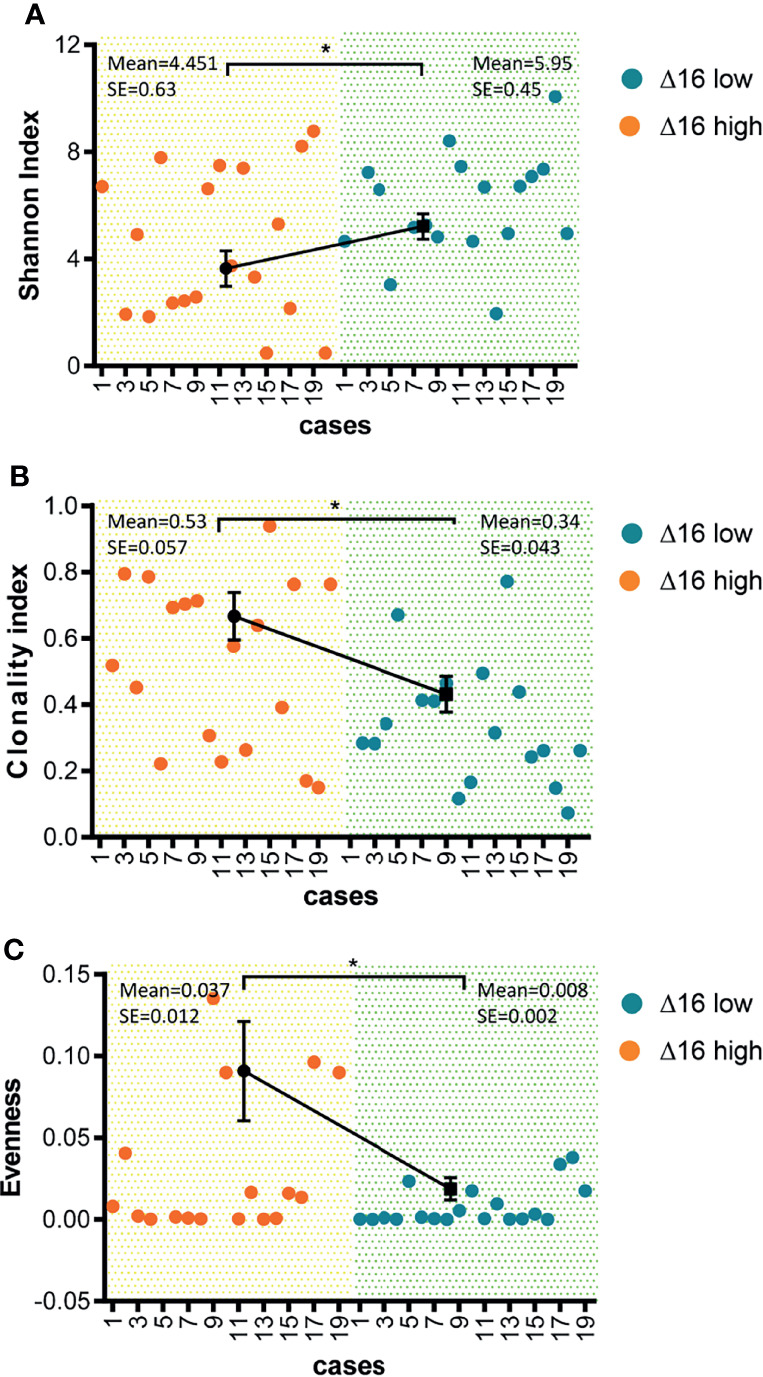
Reduced diversity of TCR clones in ERBB2d16-high patients. **(A)** Shannon index was lower in the ERBB2d16-high patients (n = 20) compared with the ERBB2d16-low patients (n = 20). **(B)** Clonality index was higher in the ERBB2d16-high patients (n = 20) compared with the ERBB2d16-low patients (n = 20). **(C)** Evenness index was higher in the ERBB2d16-high patients (n = 20) compared with the ERBB2d16-low patients (n = 20). (*p < 0.05, **p < 0.001, ***p < 0.0001). Δ16 low: ERBB2d16/ERBB2 ratio < 0.88; Δ16 high: ERBB2d16/ERBB2 ratio ≥ 0.88. *ERBB2: human epidermal growth factor receptor-2; ERBB2d16: ERBB2ΔEx16; TCR: T cell receptor*.

Through background knowledge, we had learned that neoantigens can stimulate the T cell activation of the corresponding TCR receptor. Through TCR sequencing Vβ ecological region, we can learn the diversity of TCR. And, in terms of reflecting TCR diversity, it was mainly by Shannon, that was, the more species, the higher the value and Clonality on the opposite side (22). And Evenness, it was the accumulation of the frequency from high to low, adding up to 50% of the corresponding clonotype number/total clonotype, the larger represents the more even the distribution. We sequenced 40 samples for TCR before trastuzumab therapy, the results showed that the Shannon index was significantly reduced in 20 patients of high ERBB2d16 expression compared with 20 cases of low ERBB2d16 expression (P = 0.042) (**Figure 4A**). In the ERBB2d16-high cases, clonality value increased significantly, which represented for a decrease in the number of species (P = 0.014) (**Figure 4B**). In terms of Evenness, the changes were significantly reduced in ERBB2d16-low cases. The results suggested that high ERBB2d16 expression reduced the diversity of TCR clones. (P = 0.023) (**Figure 4C**).

### Association of an Elevated ERBB2d16/ERBB2 Ratio With Less Clinical Benefit From Trastuzumab

**Figure 5:**
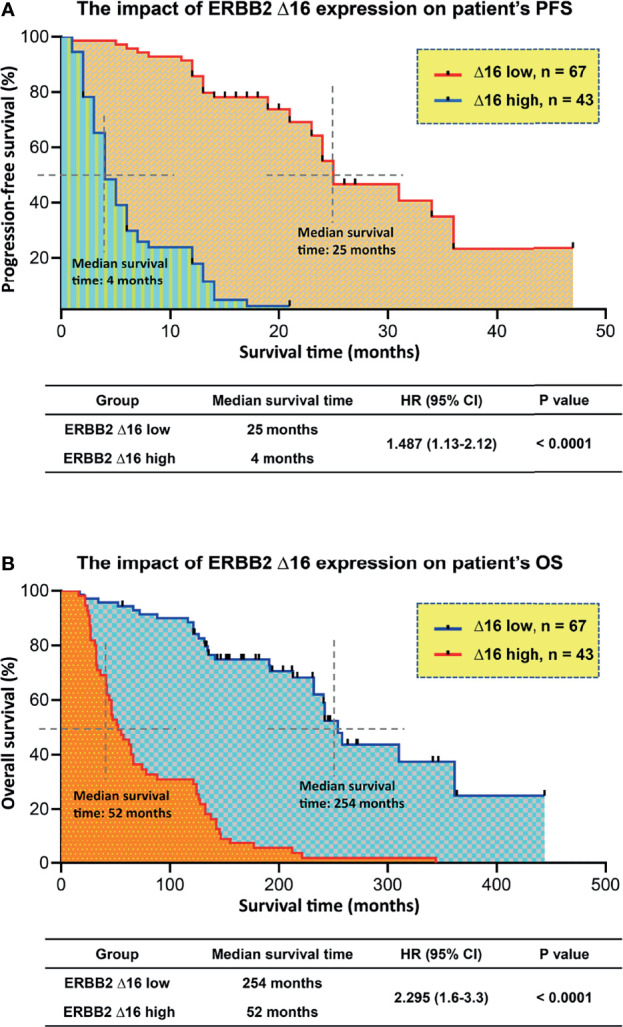
Unfavorite prognosis in patients with high ERBB2d16 expression. **(A, B)** Kaplan–Meier plotting of PFS **(A)** and OS **(B)** for patients stratified by ERBB2d16 expression who had been treated with chemotherapy plus trastuzumab (n = 110). Δ16 low: ERBB2d16/ERBB2 ratio < 0.88; Δ16 high: ERBB2d16/ERBB2 ratio ≥ 0.88. *PFS; progression free survival; OS: overall survival; HR: hazard ratio; ERBB2: human epidermal growth factor receptor-2; ERBB2d16: ERBB2ΔEx16*.

We hypothesized that tumors with high ERBB2d16 expression may be resistant to trastuzumab. Possible explanations include the lack of a juxta-membrane region in ERBB2d16 that would reduce trastuzumab binding to ERBB2d16, resulting in resistance to trastuzumab therapy (**Figure 1A**). Additionally, EMT, which is associated with high ERBB2d16 expression in tumors, may also render tumors resistant to therapy. We, therefore, evaluated the PFS from the date of initiating chemotherapy plus trastuzumab in the high and low ERBB2d16 groups. The median PFS for the low ERBB2d16 group was significantly longer than that for the high ERBB2d16 group (25 versus 4months, respectively, P < 0.0001) (**Figure 5A**). Furthermore, our results showed that the increased ERBB2d16 expression in human gastric cancer was associated with the reduced overall survival time (OS). The median OS for the low ERBB2d16 group was significantly longer than that for the high ERBB2d16 group (254 versus 52 months, respectively, P < 0.0001) (**Figure 5B**).

Cox proportional hazards models were then used to quantify the prognostic significance of risk factors after multivariable adjustment. A multivariable analysis was performed to assess the factors that demonstrated significant effects as in the univariate analysis. After adjusting for competing risk factors, high ERBB2d16 expression was identified as a risk factor in PFS (HR: 1.462; 95% CI: 1.314–1.806, P = 0.01), which means high ERBB2d16 expression increases the risk of progression or death by 46.2% compared with low ERBB2d16 expression. High vimentin expression was associated with an adverse prognosis in patients (HR: 1.364; 95% CI: 0.983– 1.843, P =0.05), which means high vimentin expression increases the risk of progression or death by 36.4% compared with low vimentin expression. Furthermore, high ERBB2d16 expression was identified as the only risk factor in OS (HR: 1.255; 95CI: 1.008-1.442, P=0.05), which means high ERBB2d16 expression increases the risk of death by 25% compared with low ERBB2d16 expression. However, E-cadherin, gender, age, TNM stage and degree of differentiation had no significant effect on PFS and OS. The details are shown in **Table 3**.

**Table 3:** Multivariable Cox proportional hazard regression analysis of patients’ demographic and clinical characteristics and survival (n = 110).

Variables	n	PFS		OS	
		HR (95%CI)	*P*-value	HR(95%CI)	*P*-value
ERBB2d16		1.462 (1.314-1.806)	0.01	1.255 (1.008-1.442)	0.05
High	43				
Low	67				
Vimentin		1.364 (0.983-1.843)	0.05	1.026 (0.843-1.524)	0.103
High	31				
Low	79				
E-cadherin		0.862 (0.833-1.083)	0.072	0.423 (0.112-0.84)	0.098
High	52				
Low	58				
PD-L1		1.033 (1.0002-2.112)	0.035	1.021 (0.911-2.312)	0.062
High	34				
Low	76				
Sex		1.071 (0.604-1.352)	0.641	1.113 (0.244-1.313)	0.77
Female	52				
Male	58				
Age		1.048 (0.827-1.282)	0.886	1.245 (0.132-2.1)	0.892
≥ 60	14				
< 60	96				
TNM staging:		1.102 (0.923-1.935)	0.697	1.099 (0.879-1.562)	0.109
IV	12				
I-III	98				
Differentiation		0.759 (0.684-1.171)	0.073	1.242 (0.782-1.293)	0.113
Well	27				
Poor	83				
No. of metastases		1.135 (0.871-1.371)	0.264	1.31 (0.988-1.882)	0.092
>1	7				
=1	5				

PFS, progression free survival; OS, overall survival; HR, hazard ratio; ERBB2, human epidermal growth factor receptor-2; ERBB2d16, ERBB2ΔEx16; PD-L1, programmed death ligand 1.

According to the above findings, we concluded that the elevated expression of ERBB2d16 was associated with the shortened survival of patients with gastric cancer and resistance to trastuzumab. In patients with locally advanced or metastatic HER2-overexpressing gastric cancer, elevated ERBB2d16 can be an independent risk factor affecting prognosis.

## Discussion

In this study we clarified that ERBB2d16 expression in HER2-positive gastric cancer was associated EMT phenotype, immunosuppression, and resistance to trastuzumab treatment. Moreover, the increased expression of ERBB2d16 might serve as an independent prognostic factor for patients with advanced HER2-positive gastric cancer.

The role of ERBB2d16 played in promoting EMT has been reported in breast cancer, and in the prior studies, the number of mesenchymal cells was significantly increased in high ERBB2d16 tumor tissues compared with high ERBB2 breast tumor tissues (7). Western blotting demonstrated that mesenchymal markers were overexpressed in high ERBB2d16 cell line compared with the high ERBB2 cell line, and some EMT transformation pathways, such as the notch pathway, that were suppressed by ERBB2 were also overexpressed in the high ERBB2d16 group. These results illustrated that ERBB2d16 may promote EMT, increase the number of circulating tumor cells, accelerate tumor metastasis, and render resistance to trastuzumab. ERBB2 exon 16 skipping is also reported to be another mechanism of TKI resistance in EGFR-mutated patients with lung cancer, in addition to its role in other solid malignancies as an oncogenic driver (23, 24). Furthermore, ERBB2d16 expression in lung cancer cells suggested that ERBB2d16 might be an oncogene in lung cancer as well (25).

In this study we used ERBB2d16/ERBB2 ratio to indicate and differentiate the expression of ERBB2d16 for the first time and found that high expression of ERBB2d16 was related to EMT and resistance to trastuzumab, which was consistent with the previous study in breast cancer.

Notably, upon multivariate analysis, the increased expression of ERBB2d16 turned out to be an independent prognostic factor of the shortened PFS and OS for patients with HER2-positive metastatic or advanced gastric cancers. Its prognostic value was even better than those of the classic pathologic factors such as TNM stage and differentiation of tissue, which suggested that the expression of ERBB2d16 might be a novel factor to predict the prognosis of patients with HER2-positive gastric cancer. It should be point out that ERBB2d16 expression has been reported to indicate benefit of trastuzumab treatment in cell experiments and 6 patients. Considering the different sample size and design across studies concerning on ERBB2d16, the value of ERBB2d16 in predicting trastuzumab treatment efficacy merits validation in well-designed prospective clinical trial (26–28). However, whether the expression of ERBB2 could be regulated by ERBB2d16 and whether ERBB2d16 can increase the therapeutic effect of trastuzumab in combination with other gene expression in some specific populations remains to be explored in the near future.

It has been reported that EMT can alter the immune microenvironment of tumors (29, 30). As indicated in these reports, mesenchymal marker expression increased, the infiltration of suppressive immune cells in the tumor microenvironment, including CD3+ CD8+ PD-1+ T cells, CD4+ FOXP3+ Tregs, and M2 macrophages, increased, and the infiltration of cytotoxic T cells decreased. PD-L1 expression also increased significantly. Based on these data and our observation of increased EMT in ERBB2d16-high tumors, we propose that the expression of ERBB2d16 might be associated with the suppressive immune microenvironment and promote the expression of inhibitory immune signals. At the gene and protein expression levels, we observed that the expression of PD-L1 increased significantly in the high ERBB2d16 expression group. The reason for the obvious increase in PD-L1 in ERBB2-overexpressing gastric cancer is that STAT3 in the ERBB2 pathway and the ERBB2d16 pathway can directly increase the expression of PD-L1 in tumor tissue, as reported (31–33). High ERBB2d16/ERBB2 expression may also promotes EMT, which increases PD-L1 expression and constitutively activates the STAT3 pathway (34, 35). However, the ramification of this increased expression of PD-1 and PD-L1 is unclear, and future studies need to evaluate whether ERBB2d16-high tumors are more or less sensitive to checkpoint blockade with anti-PD-1 or anti-PD-L1 therapies.

Some research verified that the production of tumor neoantigens can be presented to T cell receptors through MHC and cause T cell killing (11, 12). It was accepted widely that TCR clonal diversity can reflect tumor immune status. The increased TCR clonal diversity detected in peripheral blood represents for an activation of anti-tumor immunity where more tumor clone subsets could be recognized and killed by immune cells. Moreover, TCR diversity can also reflect the number of neoantigens in tumor tissues and the strength of tumor immune response (36, 37). To better understand the effect of ERBB2d16 expression on immune response, we collected patients’ peripheral blood for TCR sequencing to explore whether ERBB2d16 expression affects TCR clone diversity. The result indicated that high ERBB2d16 expression in tissues could reduce the diversity of TCR clones. Compared with low ERBB2d16 expression, high ERBB2d16 expression was significantly correlated with reduced tumor immune response and immunosuppression. At present, anti-PD-1 therapy combined with anti-HER2 treatment has been used in HER2-positive gastric cancer and has achieved good clinical effects (38). In such cases, based on the result in this study, ERBB2d16 expression verification should be performed to determine whether the patient is suitable for immunotherapy when anti-PD-1 treatment is considered for the patients with HER2-positive gastric cancer.

We have to address several limitations in our study. Firstly, the limited sample size and tumor heterogeneity might influence the reliability of conclusion. Secondly, E-cadherin and vimentin without concomitant examination of other EMT genes might reduce the accuracy of EMT assessment to some extent. However, the mesenchymal cell makers including N-cad, twist and ZEB1 were examined in partial samples using RT-PCR and the result also suggested that ERBB2d16 might promote EMT phenotype (**Supplementary Figure 1** and **Supplementary Table 1**). Similarly, in the examination of immunosuppression in microenvironment, total CD3-positive T cells rather than subtyped T cells were examined using multiple immunofluorescence staining in this study provided limited clues to figure out the complexity of microenvironment. Lastly, the mechanism underlying EMT, immunosuppression as well as resistance to trastuzumab by ERBB2d16 remains unclear.

In summary, this study found that EMT and an impaired tumor immune microenvironment resulting from high ERBB2d16 expression could influence the clinical response to trastuzumab in gastric cancer patients. These results provided new directions for ERBB2d16-targeted treatment in patients with gastric cancer in the future. For example, the combinational effect of PD-1 and PD-L1 monoclonal antibodies with inhibitors of the AKT/PI3K/mTOR signaling pathway (downstream of HER2) has been observed (10, 39) and could potentially be applied to ERBB2d16-high tumors. In the future, we will study the relationship of ERBB2d16 with other cell populations and molecules associated with immune responses, such M2 macrophages, Tregs, and MHC molecules, to identify other novel therapies for these aggressive malignancies. At the same time, we will summarize the evaluation on the efficacy of trastuzumab combined with antiPD-1 in patients with high ERBB2d16 expression.

## Data Availability Statement

The data presented in the study are deposited in the dryad repository and is available here: https://datadryad.org/stash/share/Cs1TzRScI_svVy6r2t6nGvJKHcOTwRjIWfykFfqNP_c.

## Ethics Statement

All participants provided written informed consent for the use of their specimens and clinical data in this research. The study protocol was approved by the Institutional Review Board of Beijing Shijitan Hospital Ethics Committee, and the ethics number is sjtk11-1x-2021 (34) and [2019] (SK014). All patients were treated in accordance with the Declaration of Helsinki.

## Author Contributions

All authors contributed to data analysis, drafting or revising the article, have agreed on the journal to which the article will be submitted, gave final approval of the version to be published, and agree to be accountable for all aspects of the work.

## Funding

This work was supported by the Key Science & Technology Project of Beijing Educational Committee and the Beijing Municipal Natural Science Foundation (grant number KZ202110025029), National Natural Science Foundation of China (grant number 81572799).

## Conflict of Interest

The authors declare that the research was conducted in the absence of any commercial or financial relationships that could be construed as a potential conflict of interest.

## Publisher’s Note

All claims expressed in this article are solely those of the authors and do not necessarily represent those of their affiliated organizations, or those of the publisher, the editors and the reviewers. Any product that may be evaluated in this article, or claim that may be made by its manufacturer, is not guaranteed or endorsed by the publisher.
