# Supplementary figures and images for: ERBB2D16 Expression in HER2 Positive Gastric Cancer Is Associated With Resistance to Trastuzumab
Source: Front Oncol. 2022 Apr 7;12:855308. doi: 10.3389/fonc.2022.855308 (PMC9021701; doi:10.3389/fonc.2022.855308)

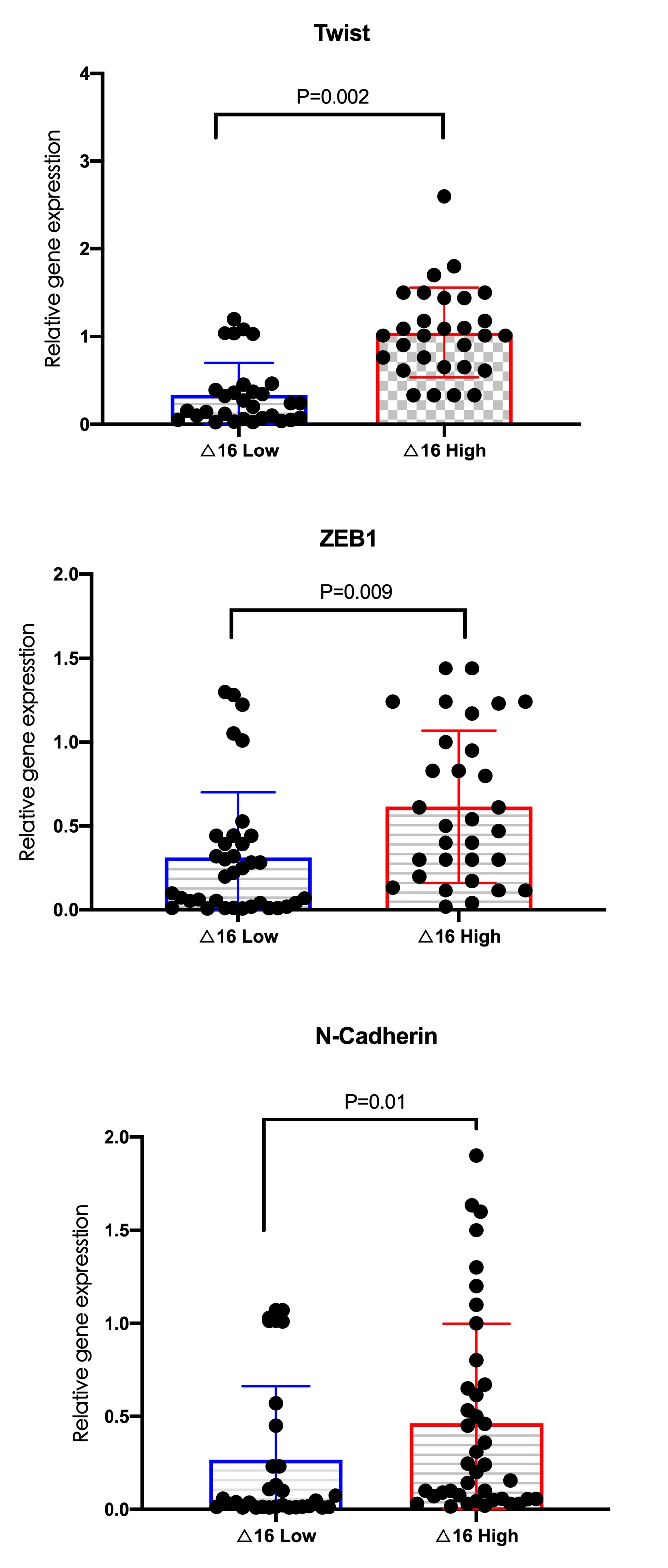

Supplement: Supplementary file 1 [file Image_1.jpeg]
