# Supplementary material for: ERBB2D16 Expression in HER2 Positive Gastric Cancer Is Associated With Resistance to Trastuzumab
Source: Front Oncol. 2022 Apr 7;12:855308. doi: 10.3389/fonc.2022.855308 (PMC9021701; doi:10.3389/fonc.2022.855308)
Supplement: Supplementary file 2 [file Table_1.docx]

| **Supplement Table 1: Prime sequence** | |  |
| --- | --- | --- |
| **Prime** | **Forward** | **Reverse** |
| N-Cadherin | 5’-TGAAACGGCGGGATAAAGAG | 5’-GGCTCCACAGTATCTGGTTG |
| ZEB1 | 5’-CAATGATCAGCCTCAATCTGCA | 5’-CCATTGGTGGTTGATCCCA |
| Twist | 5'-AGACCGAGAAGGCGTAGC | 5'-TGAGCAAGATTCAGACCC |
